# Spontaeneous subacute portomesenteric venous thrombosis: a case report

**DOI:** 10.1186/1757-1626-1-128

**Published:** 2008-08-27

**Authors:** Muhammed Mushtaque, Ronan A Cahill, John J Sheehan, Richard B Stephens

**Affiliations:** 1 Department of General Surgery, St James's Hospital, Dublin, Ireland

## Abstract

Although uncommon and often asymptomatic, portal venous thrombosis can have catastrophic consequences for the individual it afflicts, particularly when the process propagates to involve the superior mesenteric vein. Familiarity with the condition's pathogenesis and presentation however permits early diagnosis and allows aggressive conservative management to achieve a successful outcome. Here we describe the successful outcome of such management for a 42-year-old male patient who developed this condition spontaneously.

## Case Presentation

**Figure 1:**
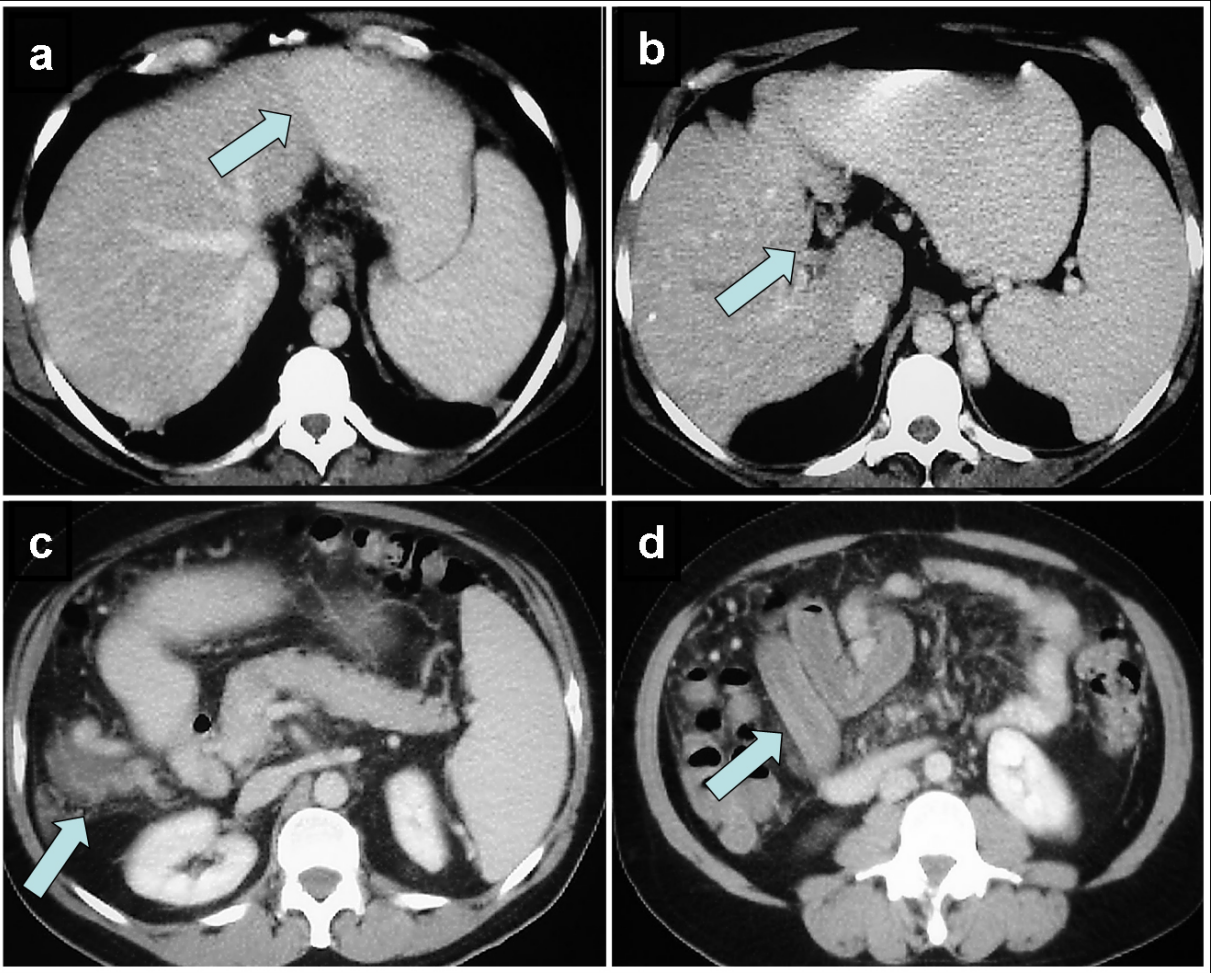
**Computerized tomographic imaging of patient's abdomen at presentation**. A computerized tomogram of the patient's abdomen performed soon after admission demonstrating (a) hypoperfusion of the right side of his liver (demarcation line indicated by blue arrow); (b) cavernous replacement of the portal vein (arrowed) consistent with thrombotic occlusion of this vessel and (c) varices around the gallbladder (arrowed). In addition the scan showed (d) a thickened loop of ileum (arrowed) suggestive of incipient venous gangrene secondary to concomitant thrombosis of the superior mesenteric vein.

**Figure 2:**
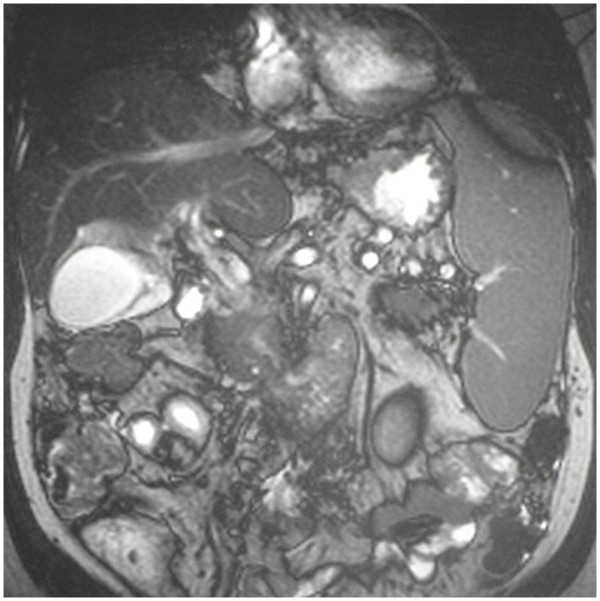
**Magnetic resonance imaging of patient's abdomen shortly after admission**. Saggital Magnetic Resonance Image showing varices around the gallbladder as well as marked splenomegaly.

A 42-year-old male Caucasian lawyer presented as an emergency with severe generalized abdominal pain of sudden onset that radiated straight through to his back without marked abdominal tenderness on examination. He also reported several episodes of vomiting but no particular aggravating or relieving factors. He was an ex-smoker of three years standing and admitted moderate alcohol consumption (25 U/week). His weight was 115 kg while his height was 195 cm (BMI = 30.2 kg/m^2^). Both his father and mother had suffered myocardial infarcts at an early age (respectively at 40 and 50 years of age). Hematological and biochemical profiling revealed a mild neutrophilia but normal amylase and troponin levels. A computerized tomogram of his abdomen demonstrated hypoperfusion of the right side of his liver (see Figure 1[a]) with cavernous replacement of the portal vein (consistent with thrombotic occlusion of this vessel, see Figure 1[b]) and varices around the gallbladder (see Figure 1[c]). In addition the scan showed a thickened loop of ileum suggestive of incipient venous gangrene secondary to concomitant thrombosis of the superior mesenteric vein (see Figure 1[d]). An MRI was also performed to further visualize these findings (see Figure 2) and to investigate the patency of the superior mesenteric vein (occluded also). The patient was immediately commenced on full therapeutic anticoagulation (intravenous unfractionated heparin) and was closely observed for signs of peritonitis. He gradually made a full recovery over the next five days. Although his thrombotic screen failed to determine the presence of any specific, inherent procoagulant tendency, he was empirically commenced on oral coumarin treatment. He remains well on follow-up after a period of six months.

## Discussion

Portomesenteric ischemia accounts for approximately 5–15% of all cases of mesenteric ischemia and has been associated with mortality rates of 20–50% [1,2]. Recent thrombosis of the portal vein may be asymptomatic or else may be associated with a systemic inflammatory syndrome with or without signs of intestinal ischemia. Old thrombosis of the portal vein is usually only recognizable on imaging by the demonstration of its cavernous transformation. Such a "portal cavernoma" refers to venous collateralization around the portal vein which develops in response to occlusion of the extrahepatic portal system and which partially maintains hepatopedal blood flow [3]. It has been previously shown that the interval between obstruction of the portal vein and the cavernous transformation is approximately 5 weeks [4]. These multiple, millimetric veins tends to occur predominantly around the suprapancreatic part of the common bile duct and may result in cholestasis due to the resulting angulation and even stenosis of the duct [5]. The main complication however of chronic portal vein thrombosis is gastrointestinal bleeding due to rupture of esophageal varices or portal hypertensive gastropathy. Although less frequent, intestinal necrosis may occur due to thrombotic extension that can result in obstruction of the superior mesenteric vein. The cause of thrombosis may be either a general prothrombotic state (e.g. myeloproliferative syndrome, antiphospholipid syndrome, antithrombin deficiency, protein C or S deficiencies, or factor gene mutations) or intraabdominal inflammation (including pancreatitis and inflammatory bowel disease). Furthermore, portal vein occlusion has been reported to occur after abdominal surgery (in particular splenectomy)[6].

Although surgery may be required when venous gangrene of the intestine occurs, early diagnosis may allow successful conservative management with anticoagulation. Although thrombolysis has been recently proposed [7], heparinization remains the first-line treatment. For this, unfractionated heparin infusion is preferable to fractionated subtypes because of its shorter half-life and ease of reversibility. Upper gastrointestinal bleeding risk can be prevented by beta-adrenergic blockade, endoscopic ligation, or endoscopic sclerosis of varices. Because the risk of disease progression persists early after initiation of therapy, a low threshold for operative exploration is required during conservative management. In the long-term, permanent anticoagulant treatment is recommended when a permanent prothrombotic state exists, even in patients who have a history of gastrointestinal bleeding.

## Consent

Written informed consent was obtained from the patient for publication of this case report and accompanying images. A copy of the written consent is available for review by the Editor-in-Chief of this journal.

## Competing interests

The authors declare that they have no competing interests.

## Authors' contributions

MM and RAC analyzed and interpreted the patient's clinical data. JS provided the radiological expertise in interpreting the images. All authors contributed to the writing of the manuscript. All authors read and approved the final manuscript.

## References

[B1] Robbins MR, Comerota AJ, Pigott JP (2005). Mesenteric venous thrombosis. Vasc Med.

[B2] Kumar S, Sarr MG, Kamath PS (2001). Mesenteric venous thrombosis. N Engl J Med.

[B3] Vibert E, Azoulay D, Castaing D, Bismuth H (2002). Portal cavernoma: diagnosis, aetiologies and consequences. Ann Chir.

[B4] Ohnishi K, Okuda K, Ohtsuki T, Nakayama T, Hiyama Y, Iwama S, Goto N, Nakajima Y, Musha N, Nakashima T (1984). Formation of hilar collaterals or cavernous transformation after portal vein obstruction by hepatocellular carcinoma. Observations in ten patients. Gastroenterology.

[B5] Condat B, Vilgrain V, Asselah T, O'Toole D, Rufat P, Zappa M, Moreau R, Valla D (2003). Portal cavernoma- associated cholangiopathy: A clinical and MR cholangiography coupled with MR portography imaging study. Hepatology.

[B6] Ikeda M, Sekimoto M, Takiguchi S, Kubota M, Ikenaga M, Yamamoto H, Fujiwara Y, Ohue M, Yasuda T, Imamura H, Tatsuta M, Yano M, Furukawa H, Monden M (2005). High incidence of thrombosis of the portal venous system after laparoscopicn splenectomy. Ann Surg.

[B7] Henoa EA, Bohannon WT, Silva MB (2003). Treatment of portal venous thrombosis with selective superior mesenteric artery infusion of recombinant tissue plasminogen activator. J Vasc Surg.

